# Relative contribution of maize and external manure amendment to soil carbon sequestration in a long-term intensive maize cropping system

**DOI:** 10.1038/srep10791

**Published:** 2015-06-03

**Authors:** Wenju Zhang, Kailou Liu, Jinzhou Wang, Xingfang Shao, Minggang Xu, Jianwei Li, Xiujun Wang, Daniel V. Murphy

**Affiliations:** 1 Ministry of Agriculture Key Laboratory of Crop Nutrition and Fertilization, Institute of Agricultural Resources and Regional Planning, Chinese Academy of Agricultural Sciences, Beijing 100081, P. R. China; 2 National Engineering and Technology Research Center for Red Soil Improvement, Jiangxi Institute of Red Soil, Jinxian 331717, P.R. China; 3 Earth System Science Interdisciplinary Center, University of Maryland, College Park, MD 20740, USA; 4 Soil Biology and Molecular Ecology Group, School of Earth and Environment, Institute of Agriculture, University of Western Australia, Crawley, WA 6009, Australia

## Abstract

We aimed to quantify the relative contributions of plant residue and organic manure to soil carbon sequestration. Using a 27-year-long inorganic fertilizer and manure amendment experiment in a maize (*Zea mays* L.) double-cropping system, we quantified changes in harvestable maize biomass and soil organic carbon stocks (0–20 cm depth) between 1986-2012. By employing natural ^13^C tracing techniques, we derived the proportional contributions of below-ground crop biomass return (maize-derived carbon) and external manure amendment (manure-derived carbon) to the total soil organic carbon stock. The average retention of maize-derived carbon plus manure-derived carbon during the early period of the trial (up to 11 years) was relatively high (10%) compared to the later period (22 to 27 years, 5.1–6.3%). About 11% of maize-derived carbon was converted to soil organic carbon, which was double the retention of manure-derived carbon (4.4–5.1%). This result emphasized that organic amendments were necessary to a win-win strategy for both SOC sequestration and maize production.

Soil organic carbon (C) is the largest terrestrial carbon reservoir and has attracted much attention because of its significance to soil fertility, food security, and climate change mitigation^1^,^2^,^3^. Increased soil organic carbon typically benefits crop production through provision of an energy source for microbial nutrient cycling and improved soil physical and chemical properties. In turn, increased crop net primary production can lead to greater above- and below-ground plant residue that can be returned to the soil, benefiting soil carbon sequestration in agro-ecosystems^4^. However, even when mineral fertilizers are applied, the carbon input from increased plant growth (returned residues and below-ground biomass) will not necessarily balance the continued decline in soil organic matter due to microbial decomposition^5^. Application of organic amendments to soil in the form of livestock manure and returned crop residues including straw is commonly recommended because of their positive effects on soil organic carbon accumulation^5^,^6^,^7^,^8^.

Soil carbon sequestration efficiency (i.e. retention rate) can be determined from the proportion of applied organic amendments that is microbially processed and retained in the soil to form new soil organic matter. Retention rates of organic amendments reported in short-term studies vary widely because of incomplete microbial decomposition^9^,^10^. Long-term field observations of soil organic carbon dynamics are now widely used to calculate retention rates of annual organic amendment inputs^10^,^11^,^12^. The retention of organic amendments is largely regulated by climate, soil properties^13^,^14^, organic amendment quantity and quality^15^, cropping sequence, and the duration of experiment^16^,^17^. A meta-analysis based on long-term experimental trials determined the average global retention of livestock manure to be 12%^6^, and tropical climates had a lower retention (7 ± 5%) of manure compared to temperate climates (23 ± 15%)^18^,^19^.

The type of organic material also influences retention rate, with more chemically resistant components such as lignin and polyphenol having higher retention rates. For example, the retention rates of livestock manure (11–23%) were much higher than that of wheat straw (3%) in a long-term study of a rice-wheat system in a subtropical climate^20^. Studies on the retention of straw-carbon following residue incorporation tend to report varying rates, even within similar climatic zones^9^,^21^ owing to the varying quality of plant residues among plant species. Furthermore, the quality of below-ground parts such as roots and root exudates, which account for 20–30% of the total assimilated carbon into soil^22^, differs from that of above-ground parts such as leaf residue. For example, in one study the retention rates of maize shoots were 5.5–6.5% but those of roots were 16–30%^23^. Long-term studies also suggest that below-ground root biomass contributes more organic carbon to soils than organic carbon incorporated in fresh leaf residues^24^,^25^. However, the retention rates of manures and various crop-derived carbon sources remain unclear.

Crop production is the primary land use in China, and covers an area of 122 million hectares^26^, accounting for 7–12% of the global soil organic carbon stock in land under arable production^27^. These agro-ecosystems are rapidly becoming mineral fertilizer intensive, and China is now the largest consumer of inorganic fertilizer in the world. Simultaneously, manure application to soil has decreased substantially in the past two decades. To quantify the impact of long-term fertilizer application on soil organic carbon dynamics and to elucidate the underlying mechanism that may sustain long-term intensive maize cropping, we studied a maize double-cropping system (spring maize and summer maize) in southern China. Based on this long-term experiment, we aimed to: (i) Investigate how long-term intensive maize double-cropping system affect soil organic carbon dynamic when managed under varying mineral fertilizer and organic manure treatments and (ii) Quantify the relative contributions of maize-derived and manure-derived carbon to soil organic carbon equilibrium and/or accumulation.

## Results

### Crop biomass

**Figure 1:**
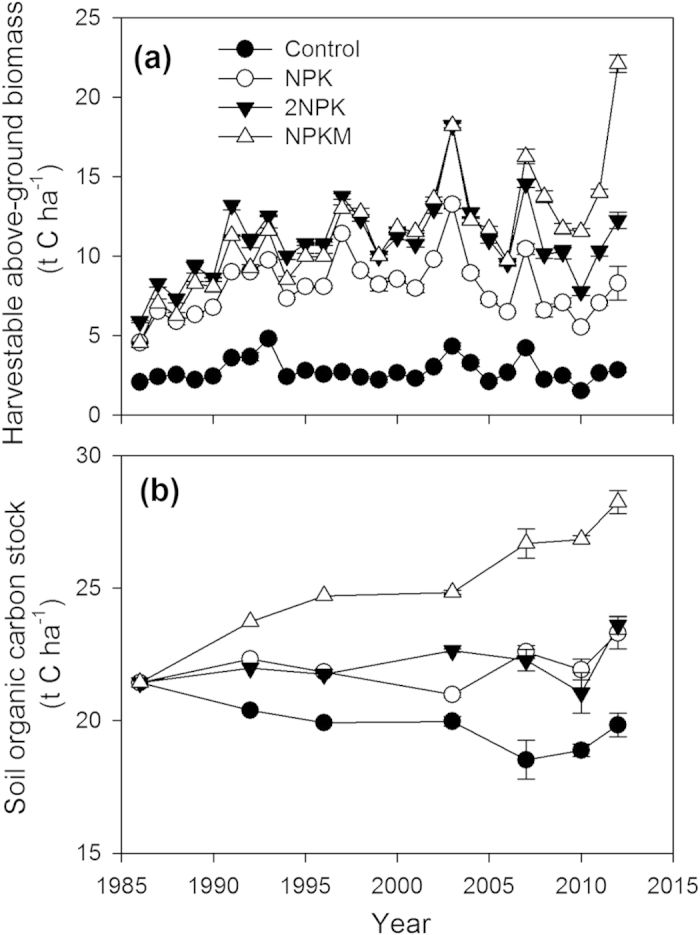
Annual harvestable above-ground biomass (t C ha^−1^) (**a**) and soil organic carbon (SOC) stock (t C ha^−1^) under with no fertilization (Control), chemical fertilizers applied at the standard rate (NPK), double application of chemical fertilizers (2NPK), and chemical fertilizers at the standard rate plus pig manure (NPKM) in a maize double-cropping system (1986–2012). Error bars for the harvestable above-ground biomass and SOC represent standard errors (SEs) of the means. For (**b**), statistics were not applied to the 1986, 1992, and 1996 data because they represent combined soil samples from three replicates for each treatment.

All fertilizer treatments showed improvements in harvestable above-ground biomass production over the course of the study (1986–2012), while there was no significant change in the control (average 1986–2012 = 2.8 t C ha^−1^ yr^−1^; Fig. 1a). Addition of extra mineral nutrients (i.e. 2NPK) resulted in significantly higher plant biomass production (LSD, *P *< 0.0001) compared to the standard (NPK) application rate. When compared to NPKM, the 2NPK treatment had significantly higher (*P* = 0.033) above-ground biomass yield for the first 10 years of the experiment (except for 1990), but had a significantly lower (*P* = 0.002) above-ground biomass yield for the last 6 years, indicating that addition of extra mineral nutrients was not a long-term replacement option for manure application. The control treatment without any fertilizer had the lowest maize biomass production.

Above- and below-ground maize-carbon allocation was quantified in 2007. In the fertilizer treatments, maize root biomass-carbon (0–20 cm depth) was 3–4 times greater than the control, and maize shoot biomass-carbon was 2–4 times greater than the control (Table 1, *P* < 0.001). The ratio of maize root to shoot (R:S ratio) was about 0.29 in the NPK treatment, whereas it was 0.19 for the other three treatments (i.e., control, 2NPK, and NPKM treatments). With the majority of the above-ground biomass removed, the remaining maize stubble accounted for 1–3% of the above-ground plant biomass (Table 1). By using a maize-root distribution correction to account for un-sampled root mass below the 0–20 cm soil collection layer^28^,^29^, we calculated that maize-derived carbon from roots plus stubble accounted for 27% of the total plant-carbon.

### Soil changes under long-term fertilizer treatments

Twenty-seven years of fertilizer and manure addition resulted in significant differences in soil nutrient content among treatments (Table 2). Treatments without manure (i.e. the control and NPK and 2NPK ) had no effect on soil total N content or soil bulk density, but decreased pH by 1–1.4 units compared to the initial year. Extra mineral fertilizer (2NPK) resulted in a further decrease in pH of 0.3 units. Extra application of mineral N, P, and K fertilizers had no significant effect on soil organic carbon, total P and K, or P availability, indicating that the NPK treatment provided insufficient nutrients for maize production.

Long-term fertilizer application significantly increased total soil organic carbon compared with the control (*P* = 0.027); the combination of manure with mineral fertilizers resulted in the highest rate of soil organic carbon accumulation over the 27-year period (R^2^ = 0.93, *P* < 0.001, Fig. 1b). Mineral fertilizer applications (NPK and 2NPK) had a significant positive effect on soil organic carbon stocks compared to the control (*P* = 0.027 for NPK and *P* = 0.023 for 2NPK). The steady soil organic carbon content in mineral fertilizer treatments shows that carbon input from maize roots and residues can compensate for the decomposition of the original soil organic matter, but not build additional soil organic matter. A marked downward trend in soil organic carbon was observed in the control treatment over the period 1986–2012.

### Changes in δ^13^C in soil organic carbon

**Figure 2:**
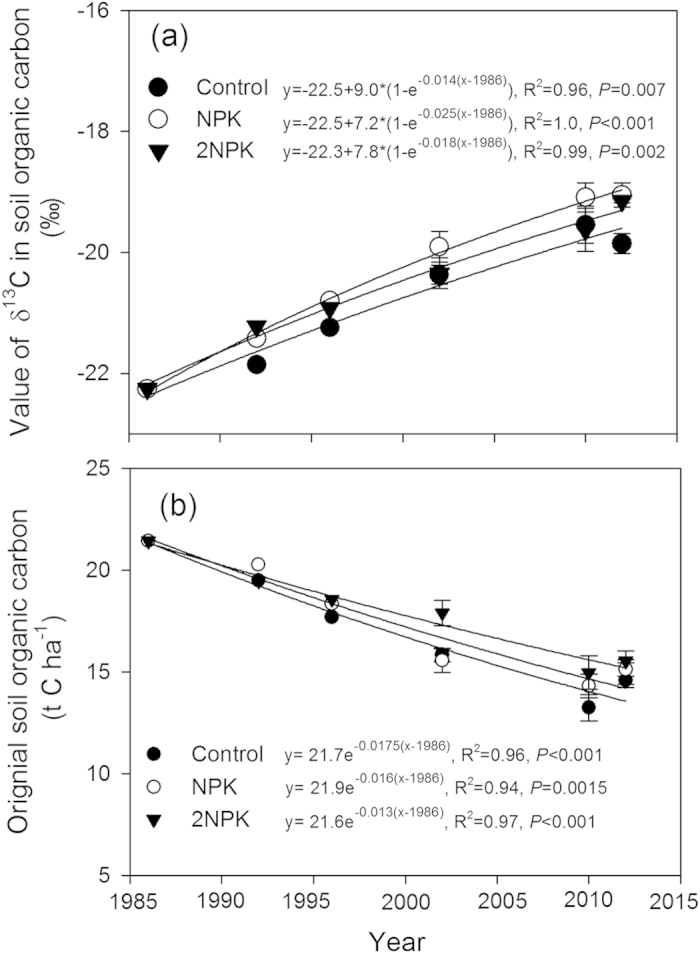
Change in δ^13^C of soil organic carbon (SOC) (**a**) and decline in the original SOC stock (**b**) under the Control and chemical fertilizer (NPK and 2NPK) treatments in a maize double-cropping system (1986–2012). Error bars for the δ^13^C and original SOC represent standard errors (SEs) of the means.

The relative abundance of δ^13^C in the soil organic carbon pool within the 0–20 cm soil layer showed a significant increase through time in the control and the mineral fertilizer treatments (NPK and 2NPK; *P* = 0.002, see Fig. 2a), indicating continual replacement of original soil organic carbon with maize-derived carbon. In the control and NPK treatments, significantly higher root biomass (*P* < 0.001; Table 2) resulted in a higher rate of increase in δ^13^C enrichment (Fig. 2a). Soil organic carbon δ^13^C increased from −22.2‰ to −19.0‰ in the mineral NPK and 2NPK treatments (Fig. 2a).

Regression analysis showed that the original soil organic carbon pool (i.e. the pool present before the experiment started) decayed at about 0.016–0.17 yr^−1^ in the control and NPK treatments, whereas the decay rate was 0.013 yr^−1^ for the 2NPK treatment (Fig. 2b). This equated to a soil organic carbon mean residence time (MRT) of 59 years for the control, whereas the MRT was 62 and 77 years for the NPK and 2NPK treatments, respectively.

### Changes in maize-derived and manure-derived soil organic carbon

**Figure 3:**
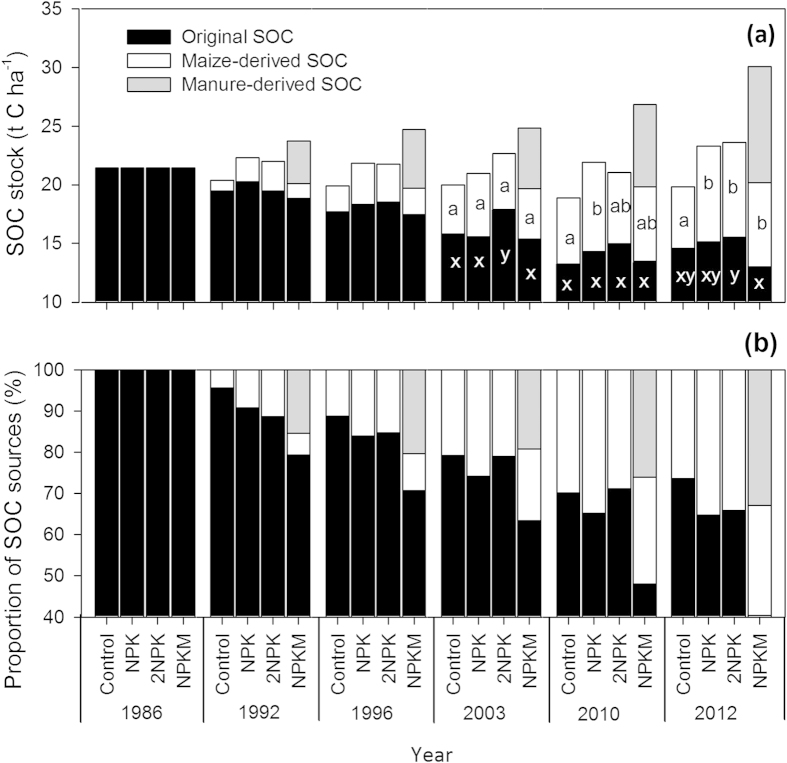
Changes in stock of original, maize-derived, and manure-derived soil organic carbon (SOC) (t C ha^−1^) (**a**) and their proportions (**b**) from 1986 to 2012 in the long-term fertilization experiment in Jiangxi province, China. The same letter indicates no significant difference at P < 0.05 for each SOC source. Statistics were not applied to the 1986, 1992, and 1996 data because they represent combined samples of three replicates for each treatment.

In the control treatment (initial soil organic carbon content = 21.43 t C ha^−1^), maize-derived soil organic carbon accumulated to 2.2 t C ha^−1^ over the first 10 years of the experiment, and eventually reached 5.6 t C ha^−1^ after another 17 years; a rate of change in the range of 0.20–0.22 t C ha^−1^ yr^−1^ (Fig. 3a). With mineral fertilizer application (NPK and 2NPK), maize-derived soil organic carbon accumulated to 3.2–3.5 t C ha^−1^ over the first 10 years and reached 8.2 t C ha^−1^ in 2012; a rate of change of 0.30 t C ha^−1^.

After 10 years, about 11% of original soil organic carbon had been replaced with maize-derived carbon in the control, whereas this was 15–16% in the NPK and 2NPK treatments (Fig. 3b). After 27 years of maize double-cropping, 26% of soil organic carbon had been replaced by maize-derived carbon in the control, and this value was 34–35% in the mineral fertilizer treatments. In the NPKM treatment, manure-derived soil organic carbon comprised about 30% of total soil organic carbon and original soil organic carbon accounted for 43%, with the remainder derived from maize.

### Maize-derived carbon and manure-derived carbon retention

**Figure 4:**
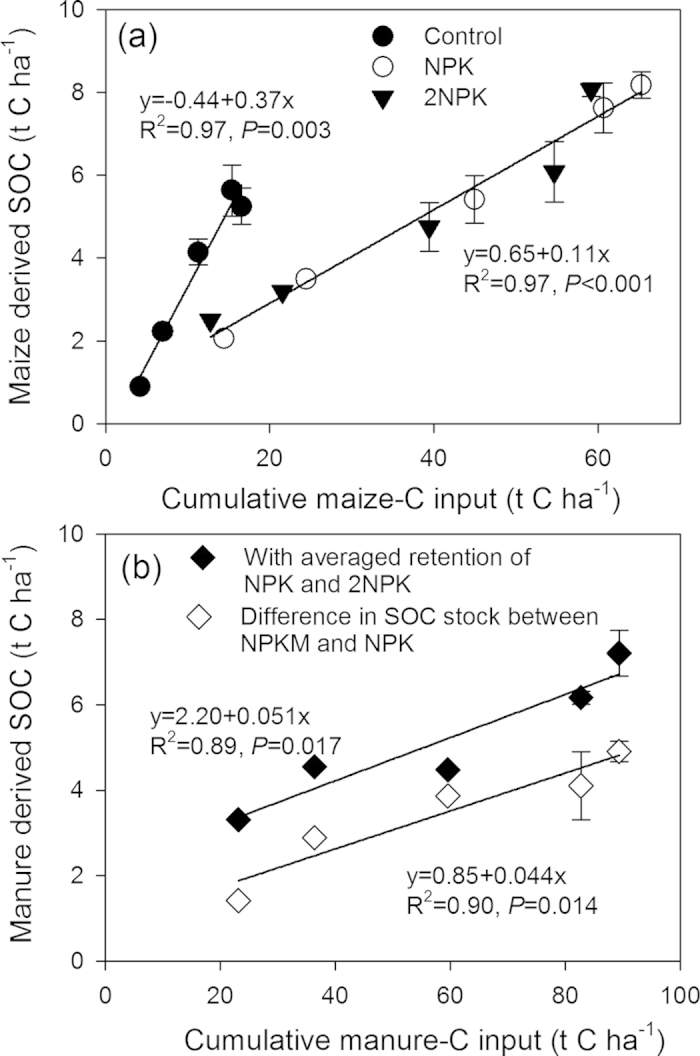
Relationship between maize-derived soil organic carbon (SOC) and cumulative maize carbon input as determined by δ^13^C measurements for the Control, NPK, and 2NPK treatments (**a**) and manure-derived SOC and cumulative manure-C inputs as determined by the regression equations in Fig. 3(a) for NPK and 2NPK treatments and by the difference in SOC stock between NPKM and NPK treatments (b).

With continual input of maize-derived carbon via incorporation of root biomass and associated root exudates into the soil, there were significant positive linear correlations between maize-derived soil organic carbon and cumulative maize-carbon input in both the control and mineral fertilizer treatments (Fig. 4a). The proportion of maize-derived carbon retained was about 37% in control soil (which had the lowest total soil organic carbon), and 11% in soil from the mineral fertilizer treatments. This result showed that the retention rate of maize-derived carbon in mineral fertilizer treatments was only about one-third of that in the control. Assuming the priming effect of long-term continuous organic manure input on original soil organic carbon turnover was insignificant^11^,^30^, our results indicate that about 5.1% of manure-derived carbon was converted into soil organic carbon (Fig. 4a). An alternative calculation, based on estimations of the difference in total soil organic carbon stock between NPKM and NPK treatments, gave a similar retention rate (4.4%) for manure-derived carbon (Fig. 4b).

**Figure 5:**
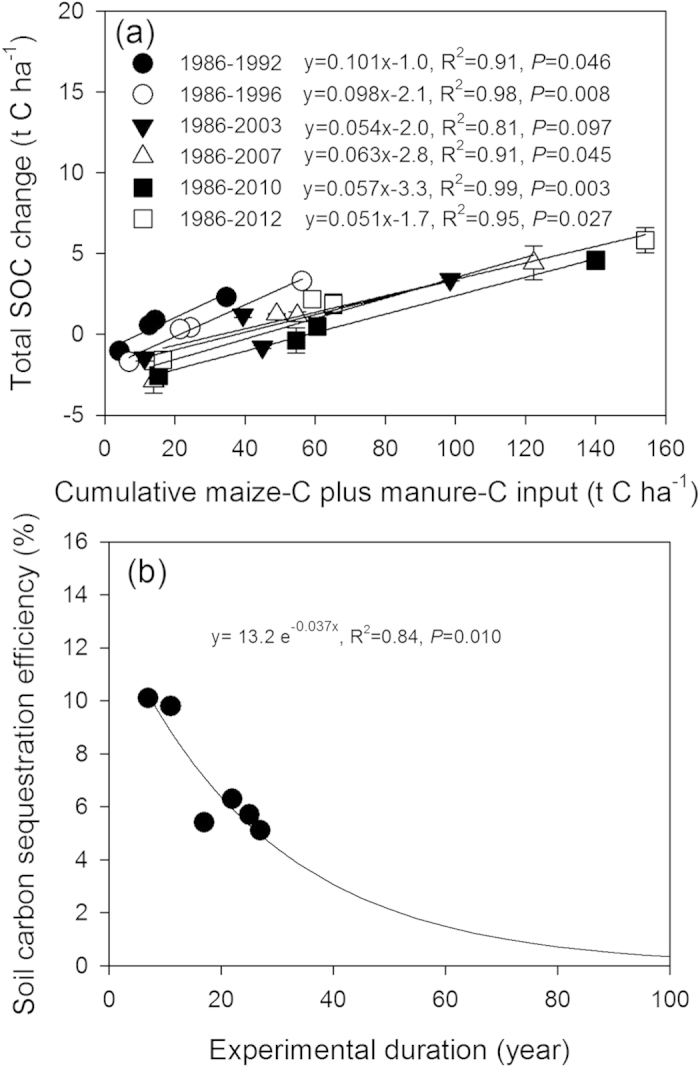
Relationships between total soil organic carbon (SOC) and cumulative maize-derived C plus manure-derived C input for the periods 1986–1992, 1986–1996, 1986–2003, 1996–2007, 1986–2010, and 1986–2012.

There was a positive, linear, and significant relationship between cumulative carbon input (i.e. maize-derived carbon plus manure-derived carbon) and increases in soil organic carbon stock (*P* < 0.045, Fig. 5a), except for the period 1986–2003, which was due to a large variation in soil organic carbon stocks between treatments in 2003 (Fig. 1b). The average retention (i.e. slope of the regression) of the maize-derived carbon plus manure-derived carbon over the first 11 years of the trial was relative high (10%) compared to over the full 27 years (5.1–6.3%); regression analysis showed that the retention rate declined significantly with time (*P* = 0.010, Fig. 5b). The average retention rate of maize-derived carbon plus manure-derived carbon was about 5.1% over the period 1986–2012.

## Discussion

Our findings illustrate that the combination of mineral fertilizers with organic manure not only improved plant biomass production, but also increased soil carbon sequestration. The marked depletion in soil organic carbon and lower crop biomass production in the control plots highlights that intensive maize cropping without the use of mineral fertilizers is not sustainable for soil fertility in this region. Additionally, soil pH has important impacts on crop productivity via direct and indirect effects on soil microbes, which are a key driver of soil nutrient cycling. Soil pH is closely related to microbial and enzyme activity^31^, which controls nutrient availability for crops. In addition, soil pH can affect soil microbe community composition and diversity^32^. We recommend the application of organic amendments, not only to improve fertility and production, but also to enhance sustainable crop production with reduced risks of soil acidification in this highly-weathered soil.

Our finding that the root:shoot ratio was higher in the standard NPK treatment than other treatments indicate that soil nutrients derived from long-term fertilizer application might have an impact on the allocation of crop-derived carbon. A similar difference in the root:shoot ratio was also observed in an organic and inorganic systems^33^. In this study, this was likely due to a decrease in the availability of nitrogen (N) and phosphorus (P) causing an increase in the relative mass of roots^8^,^34^, which resulted in a larger plant root system to uptake nutrients. This indicates that the applied mineral N, P, and K fertilizers were insufficient for maize double-cropping. In addition, below-ground carbon allocation tends to decrease with N fertilizer inputs^35^, because of stronger competition of roots for nutrients when compared with shoots, particularly at low nutrient levels^36^. Annual records of crop performance also confirm that the root:shoot ratio has a significant relationship with maximum plant height among 43 plots^37^. Our study highlights differences in root-carbon input among arable systems subjected to different fertilizer regimes, and hence a need for caution when estimating root biomass from the root:shoot ratios.

Our estimate of average retention of manure-derived carbon plus maize-derived carbon was about 5–10%, which is consistent with values reported for warm temperate areas with double-cropping system^14^. This was also in the expected range (2–13%) for tropical climates^18^,^19^. We found that the retention (11–37%, average 24%) of maize-derived carbon was in a similar range to that for maize residues and roots (5.5–16.6%) reported by Collins *et al*.^30^ in the maize belt in the USA, and that of the residue including roots (21–37%) after 11–13 years of continuous maize production^38^,^39^. Plénet *et al*.^23^ also reported a carbon retention (16–30%) for maize roots. Our estimated values of the retention of maize-derived carbon, which was mainly below-ground root biomass, is a little lower than those obtained with silage maize straw or maize straw-derived manure. The high retention rate of maize-derived carbon in the control might be in part due to the physical and chemical protection by soil clay particles acting to reduce soil organic carbon depletion as well as accumulation^40^.

Our findings indicate that retention of manure-derived carbon is about half (4.4–5.1%) that of maize-derived carbon (mainly retained from below-ground biomass, 11%) in the mineral fertilizer treatments. This indicates that, at the recommended rates of mineral fertilizer and manure application, the maize-derived carbon from below-ground crop biomass may have double the contribution of that from manure to soil carbon sequestration. Our result supports the argument of Buyanovsky and Wagner^41^ that there is no reason to consider that manure was more effective than plant residues for soil organic carbon enhancement.

We attribute the relatively high retention of maize-derived carbon in this study in part to the difference in oxygen availability as below-ground plant roots and surface applied manure decompose. We also suggest the higher retention of maize-derived carbon may be due to the following reasons: (i) the greater biochemical recalcitrance of root litter than manure-derived carbon—*in situ*^13^CO_2_ labelling studies have shown greater retention of root-derived-carbon, as occluded particulate organic carbon associated with the clay and silt fraction than manure residue-derived carbon^42^,^43^; (ii) stronger physical-chemical protection of root-derived carbon which breaks down in the soil matrix^40^ compared to manure applied at the surface and exposed to the atmosphere; and (iii) the continuous and repeated nature of maize-derived carbon inputs from root exudates and dead and living root turnover in two maize grow season annually, while manure was applied twice each year.

We also quantified the uncertainties in our estimates of the root:shoot ratio. Published values of the root:shoot ratios, commonly used to estimate carbon input in soil organic carbon modelling, typically range from 0.22^44^ to 0.26^45^ but are reported as high as 0.35^46^. We used a range from 0.22 to 0.35 to conduct the uncertainty analysis for maize-carbon retention. In the mineral fertilizer treatments, maize-derived carbon retention ranged from 7.8 to 12.5%, whereas manure-derived carbon retention ranged from 4.9 to 5.0%, i.e. twice as much maize-derived carbon was sequestered to the soil as manure-derived carbon in this study.

The significant negative relationship between the retention of total carbon (maize-derived plus manure-derived) and time in our study indicates that less carbon can be sequestered into soil after a relatively long-term annual manure application. This fits well with the concept of soil carbon saturation^47^ and likely duration of soil organic carbon sequestration^16^.

Our findings suggest that the addition of extra mineral fertilizer (2NPK) slowed soil organic carbon decomposition. This implies that adequate mineral fertilizer application is necessary to stabilize and maintain soil organic carbon levels in intensive maize cropping systems. Furthermore, our findings indicate that insufficient soil nutrients, especially N and P, limit the build-up of soil organic carbon.

## Methods

### Study site and fertilization experiment

The long-term experimental field trial was started in 1986 and is located in Jinxian county, Jiangxi province, in southern China (28°37’N, 116°26’E). The climate is monsoonal subtropical with an average annual temperature of 17.7 °C and an average annual rainfall of 1727 mm. The red soil of the study site, developed from Quaternary red clay, is classified as a Ferralic Cambisol (FAO) and Ultisol (US soil classification) and is typical of the region. Initial (1986) soil physiochemical properties are reported in (Table 2).

The study site mostly contained shrubs (*Pinus* spp. until the 1960s, when it was planted with peanuts (C3, *Arachis hypogaea*) and soybean (C3, *Glycine max* L.). In 1986, it was converted to a maize double-cropping (spring and summer maize) C4 system. The field experiment was conducted based on a randomized complete block design with three replicates of each treatment. Four fertilization treatments were included: (1) no-fertilizer (control); (2) mineral nitrogen (N), phosphorus (P), and potassium (K) at the standard application rate (NPK); (3) double the standard application rate of NPK (2NPK); and (4) the standard application rate of NPK fertilizer in combination with pig manure (NPKM). Each replicate treatment was randomly allocated to one of twelve 22.2 m^2^ plots; these were physically isolated from each other by 100-cm-thick cement baffles.

Mineral N, P, and K fertilizers were applied in the forms of urea, calcium super-phosphate, and potassium chloride, respectively. For the NPK and NPKM treatments, mineral N, P, and K fertilizers were applied to each maize crop at rates of 60 kg ha^−1^ yr^−1^, 13 kg ha^−1^ yr^−1^, and 50 kg ha^−1^ yr^−1^, respectively. For the 2NPK treatment, mineral N, P, and K were applied to each maize crop at doubled rates used in the NPK treatment. For the NPKM treatment, pig manure was applied prior to each maize crop at 15 t ha^−1^ (fresh weight). The organic carbon, total N, and P content of the pig manure were 376.1, 33.14, and 23.77 g kg^−1^ (dry matter); and the water content of pig manure was 716 g kg^−1^ (fresh matter), respectively. P fertilizer and manure were applied before maize sowing. Half of the N, and all of the K fertilizers were applied 1 week after sowing as a top dressing, with the remainder of the N fertilizer applied 2 weeks after sowing.

Spring and summer maize were sown by hand in strips in the middle of April and late July, and harvested in early July and early November, respectively. Maize was grown in rows spaced 30 cm apart, with seeds planted every 50 cm. The hybrid maize variety planted was changed every 5 years to match best practice for the region. Herbicides and pesticides were applied as required. Grain and straw were harvested manually by cutting and then air-dried, threshed, and oven dried at 70 °C to constant weight. Plant biomass and yield from each field plot were recorded annually. Field preparation, fertilizer application, irrigation, and weed control were carried out manually. Conventional tillage were performed with the aid of buffalo. All the harvestable above-ground biomass was removed from the plots, leaving stalks about 5–10 cm high.

### Soil sampling and analysis

Soil samples (0–20 cm depth) from each plot were collected in 2003, 2007, 2010, and 2012, after summer maize was harvested. For each plot, 5–10 randomly located soil cores were taken using a 5-cm-diameter auger; these were, mixed thoroughly, and air-dried for 7 days. Air-dried soil was sieved through screens: 2.0 mm for available nutrient analyses and 0.15 mm for total nutrient analyses. The sieved soil was stored in sealed plastic jars prior to analysis. Soil properties from 2012 are presented in Table 1. For 1986, 1992, and 1996, only pre-existing soil samples for each treatment were available (i.e. samples from replicate plots had been mixed). These were re-analysed to determine soil organic carbon for these years.

Soil organic carbon content of soil samples was determined by dry combustion with a CN analyzer (EA3000, Milan, Italy). Stable isotope^13^C analysis was conducted on all soil samples without added organic manure (i.e. the control, NPK, and 2NPK), except for in 2007, by using an Isoprime MAT Delta Plus XL (Bremen, Germany) at the Stable Isotope Laboratory of the Chinese Academy of Agricultural Sciences (Beijing, China). Because of the documented negligible year-to-year variation in *δ*^13^C values of plant residues^48^, maize residues were only analysed once for *δ*^13^C, in 2010.

Roots in the 0–20 cm soil layer were collected by washing soil from three randomly selected quadrats (60 × 20 cm) per plot through a 50-μm sieve. This below-ground biomass was used to calculate the root:shoot ratio in year 2007. Standing stalk residues were also collected from each plot to estimate above-ground maize-derived carbon subsequently incorporated into the soil. All plant biomass samples were oven-dried at 40 °C to constant weight, and then weighed separately to calculate the ratio of root biomass and residues incorporated into the top soil to the harvestable above-ground biomass. Organic carbon content of grain, straw, collected materials (e.g., root and stalk residues), and pig manure was 471, 451, 450, and 376 g kg^−1^ (dry matter), respectively.

### Retention rates of maize- and manure-derived carbon

To quantify the long-term retention of maize- (mainly root) and manure-derived carbon, we first calculated maize-derived carbon biomass, carbon input, and soil organic carbon as follows.

### Carbon biomass and carbon input

The annual harvestable crop biomass (in carbon, C_biomass_) was calculated according to harvestable above-ground yields of grain and straw (Y_grain_ and Y_straw_, t ha^−1^) and their respective carbon content (C_grain_, and C_straw_, kg kg^−1^).


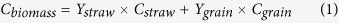


Maize-derived carbon input (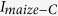) into the soil was calculated as follows:


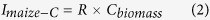


where R is the R:S ratio after correction to account for the distribution of the root system within the topsoil (i.e. only 85.1% occurs in the 0–20 cm layer^28^,^29^). Extra standing stalk that remained on the soil surface was also included in the estimation of carbon input into soil. There was about 1-3% of harvestable biomass measured as standing stalk and included in the calculation of maize-derived carbon input (Table 2).

The cumulative maize-carbon input (*CI*_*maize−C*_) was summarized for each year at the given durations:


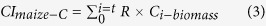


### Sources of soil organic carbon

The contribution of maize-derived carbon (below-ground roots and residues) to soil organic carbon can be calculated from natural ^13^C abundance by using a two-end member mixed model^30^,^49^. In this study, maize-derived soil organic carbon *P*_*Maize−C*_ was determined as follows:


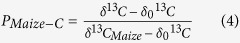


where δ^13^C and δ_0_^13^C are the stable natural abundances of ^13^C in soil organic carbon during and prior (1986) to the experiment, respectively, and δ^13^C_maize_ is the mean δ^13^C value of maize residues and roots (−13.16‰). The value of δ_0_^13^C in soil organic carbon in 1986 (prior to the commencement of the maize field trial) was −22.26‰.

For the treatments without organic manure input (i.e. the Control, NPK, and 2NPK), the maize-derived soil organic carbon was calculated as follows:


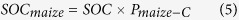



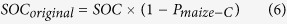


where *SOC* is total soil organic carbon.

Accordingly, a first-order kinetics model was fitted to simulate the remains of the original source according to SOC_0_ as follows:


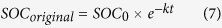


where SOC_0_ is the soil organic carbon stock prior to the experiment, and *k* is the decomposition rate of original soil organic carbon. Then, for the NPKM treatment, manure-derived soil organic carbon was estimated as the difference in total soil organic carbon and the sum of original and maize-derived soil organic carbon.

### Retention rates of maize- and manure-derived carbon

Assuming that the priming effect of continuous manure addition on original soil organic carbon decomposition is insignificant^50^,^51^, the retention rates of maize-derived carbon and manure-derived carbon were estimated by fitting regressions of maize- and manure-derived soil organic carbon against the cumulative maize- or manure-carbon input^11^,^14^. Maize-derived carbon retention was calculated from the slope of the regression equation fit to data at several intervals from treatments without manure application. For the NPKM treatment, soil organic carbon included original, maize-derived, and manure-derived sources. Manure-derived soil organic carbon was estimated using two approaches. First, we assumed that the priming effect of manure carbon additions on soil organic carbon turnover was insignificant. Maize-derived soil organic carbon was estimated using the maize-derived carbon retention rate for the NPK treatment and the accumulated maize-derived carbon input in the NPKM treatment. Original soil organic carbon was calculated according to the decomposition rate (*k*) of the original soil organic carbon in the NPK treatment and the total stock of soil organic carbon in the NPKM treatment. Therefore, manure-derived soil organic carbon was calculated as the difference between total soil organic carbon and the sum of maize-derived soil organic carbon and original soil organic carbon. Second, we estimated the change in soil organic carbon through time by calculating the difference in soil organic carbon between the NPKM and NPK treatments^52^. The total retention rate of maize-derived carbon and manure-derived carbon was then determined from the regression between the rate of change in soil organic carbon stock (1986–2012) and the cumulative carbon input (i.e. maize-derived carbon plus manure-derived carbon) among the four treatments.

### Statistical analysis

Soil organic carbon and collected maize biomass, as well as soil organic carbon δ^13^C, were analysed using one-way ANOVA in Statistical Product and Service Solutions. Linear and/or non-linear regression was employed to determine relationships between soil organic carbon sources and year or cumulative maize-derived carbon/manure-derived carbon input under different fertilizer treatments.

## Additional Information

**How to cite this article**: Zhang, W. *et al*. Relative contribution of maize and external manure amendment to soil carbon sequestration in a long-term intensive maize cropping system. *Sci. Rep*. **5**, 10791; doi: 10.1038/srep10791 (2015).

## Figures and Tables

**Table 1:** Biomass of maize root, stubble, and harvestable biomass in the topsoil (0–20 cm) and the root:shoot ratio (R:S) of maize under four fertilization treatments in a maize double-cropping system from the long-term fertilization experiment in Jiangxi province, China.

Treatment	Root biomass (t C ha^−1^)		Shoot biomass (t C ha^−1^)		Root: shoot ratio (Ac/(B+C))	Standing stabble biomass to harvestable biomass ratio (C/B)
	Measured A	Corrected* Ac	Harvestable B	Stubble C		
Control	0.70 ± 0.13 b	0.82	4.21 ± 0.04 d	0.15 ± 0.06 ab	0.19 b	0.036
NPK	2.60 ± 0.24 a	3.05	10.46 ± 0.33 c	0.12 ± 0.05 b	0.29 a	0.014
2NPK	2.40 ± 0.21 a	2.82	14.56 ± 0.25 b	0.20 ± 0.02 ab	0.19 b	0.011
NPKM	2.72 ± 0.31 a	3.20	16.27 ± 0.45 a	0.30 ± 0.07 a	0.19 b	0.018

^*^The root biomass was corrected using a percentage of 85.1% as the proportion of root biomass in the 0–20 cm soil layer (Li *et al*., 1992, Liu and Song, 2007). Different letters in the same column indicate significant differences at P < 0.05.

Maize biomass was measured in 2007.

**Table 2:** Soil properties in the topsoil (0–20 cm) in 1986 and 2012 from the long-term experimental site in Jiangxi province, China.

Year	Treatments	Total SOC (g kg^–1^)	Total N (g kg^–1^)	Total P (g kg^–1^)	Total K (g kg^–1^)	Olsen-P (mg kg^−1^)	Available K (mg kg^−1^)	Soil pH	Bulk density (g cm^−3^)
1986		8.93	0.98	0.62	11.36	5.6	69.9	6.00	1.20
2012	Control	7.69 ± 0.17 a	0.94 ± 0.02 a	0.61 ± 0.03 a	14.38 ± 0.03 a	11 ± 0 a	105 ± 13 a	4.97 ± 0.06 b	1.31 ± 0.03 a
	NPK	9.03 ± 0.24 b	0.97 ± 0.04 a	0.75 ± 0.06 ab	16.06 ± 0.38 b	21 ± 5 a	202 ± 18 b	4.91 ± 0.09 b	1.30 ± 0.05 a
	2NPK	9.15 ± 0.16 b	0.97 ± 0.02 a	0.90 ± 0.08 b	16.42 ± 0.28 b	35 ± 8 a	263 ± 4 c	4.64 ± 0.13 a	1.25 ± 0.01 a
	NPKM	11.65 ± 0.71 c	1.23 ± 0.05 b	1.87 ± 0.00 c	16.54 ± 0.20 b	221 ± 21 b	342 ± 12 d	6.12 ± 0.04 c	1.32 ± 0.01 a

Different letters in the same column indicate significant differences at P < 0.05.
